# Anti-Aging Potential of Phytoextract Loaded-Pharmaceutical Creams for Human Skin Cell Longetivity

**DOI:** 10.1155/2015/709628

**Published:** 2015-09-10

**Authors:** Saima Jadoon, Sabiha Karim, Muhammad Hassham Hassan Bin Asad, Muhammad Rouf Akram, Abida Kalsoom Khan, Arif Malik, Chunye Chen, Ghulam Murtaza

**Affiliations:** ^1^Department of Natural Resources Engineering and Management, University of Kurdistan, Hewler 44003, Iraq; ^2^University College of Pharmacy, University of the Punjab, Lahore 54000, Pakistan; ^3^Department of Pharmacy, COMSATS Institute of Information Technology, Abbottabad 22060, Pakistan; ^4^Department of Pharmacy, University of Sargodha, Sargodha 40100, Pakistan; ^5^Department of Chemistry, COMSATS Institute of Information Technology, Abbottabad 22060, Pakistan; ^6^Institute of Molecular Biology and Biotechnology, University of Lahore, Lahore 54000, Pakistan; ^7^Key Laboratory of Biorheological Science and Technology, Chongqing University, Chongqing 400030, China

## Abstract

The exposure to ultraviolet radiations (UVR) is the key source of skin sunburn; it may produce harmful entities, reactive oxygen species (ROS), leading to aging. The skin can be treated and protected from the injurious effects of ROS by using various pharmaceutical formulations, such as cream. Cream can be loaded with antioxidants to quench ROS leading to photo-protective effects. Moreover, modern medicines depend on ethnobotanicals for protection or treatment of human diseases. This review article summarizes various *in vivo* antioxidant studies on herbal creams loaded with phyto-extracts. These formulations may serve as cosmeceuticals to protect skin against injurious effects of UVR. The botanicals studied for dermatologic use in cream form include *Acacia nilotica, Benincasa hispida, Calendula officinalis, Camellia sinensis, Camellia sinensis, Nelumbo nucifera, Capparis decidua, Castanea sativa, Coffea arabica, Crocus sativus, Emblica officinalis Gaertn, Foeniculum vulgare, Hippophae rhamnoides, Lithospermum erythrorhizon, Malus domestica, Matricaria chamomilla* L., *Moringa oleifera, Morus alba, Ocimum basilicum, Oryza sativa, Polygonum minus, Punica granatum, Silybum marianum, Tagetes erecta* Linn., *Terminalia chebula, Trigonella foenum-graecum*, and *Vitis vinifera*. The observed anti-aging effects of cream formulations could be an outcome of a coordinating action of multiple constituents. Of numerous botanicals, the phenolic acids and flavonoids appear effective against UVR-induced damage; however the evidence-based studies for their anti-aging effects are still needed.

## 1. Etiologies and Types of Human Skin Aging

**Figure 1:**
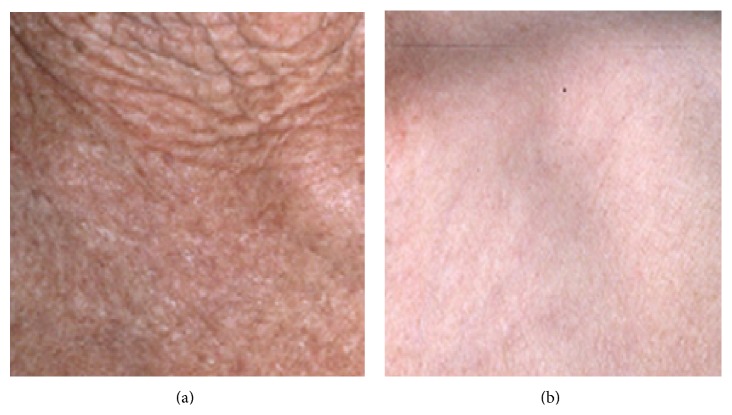
Clinical appearance of extrinsic (a) and intrinsic (b) aging of skin.

Skin aging is a dermatologic change that progresses as a person ages or is exposed to ultraviolet radiations (UVR) if no treatment is adopted. The extensive research activities are focused on this skin concern that involves the appearance of unpleasant, observable marks on skin surface due to proteolysis of cutaneous elastic fibers resulting in the reduced cell functions [1]. Skin aging can be divided into two types, that is, intrinsic aging or chronological aging (inevitable phenomenon) and extrinsic or premature or photoaging (evitable phenomenon) owing to the physiological and environmental factors, respectively [2–4]. Morphologically, photoaging is characterized by dry, rough, pigmented, and abraded skin especially of face and hands in individuals who live in sunny geographical regions and are chronically exposed to direct sunlight (Figure 1). Conversely, fine, smooth wrinkles on dry, pale skin impart the characteristics of intrinsic aging [1]. Diagnostically, intrinsic skin aging is identified by seborrheic keratosis which is not a biomarker of photoaging [5]. Pathologically, the photodamaged skin shows vascular damage that is absent in intrinsically aged skin. An increased skin vascularization and angiogenesis are observed in the photoaged skin [6]. Microscopically, thicker epidermis is another feature of the photoaged skin [4]. It is noteworthy that the strength and resiliency of skin depend on proper and uniform arrangement of collagen (types I and III) fibrils and elastin in the dermis [7]; thus, collagen deficiency may result in skin aging due to the production of collagenase and thymine dimer in skin on exposure to UVR. Histologically, the extracellular matrix of intrinsically aged skin possesses diminished levels of elastin [2], while the elastin amassing in the photoaged skin is observed just below the dermal-epidermal junction [8]. Elastin is a fibrous protein that is reduced in thickness from deeper to superficial dermis. It provides natural elasticity and strength to human body. It also plays a role in tissue repair [9]. The basic and the major molecular unit involved in the construction of human skin is collagen that is produced from procollagen. Collagen is a protein that is present in the connective tissues of human body. The dermal fibroblasts generate the procollagen under the effect of transforming growth factor-*β* (TGF-*β*) and activator protein-1 (AP-1), where TGF-*β* and AP-1 govern the production and breakdown of collagen, respectively. Under the effect of UVR received from sun, the upregulation of matrix metalloproteinases (MMPs) enzymes secreted by keratinocytes, fibroblasts, and other cells promotes breakdown of collagen by AP-1 as well as decrease in collagen synthesis (Figure 1) [10, 11]. It results in breakdown of the connective tissues during photoaging [12–14]. During adulthood, there is about 1% decrease in collagen content per year, but this rate is higher in the aged people since old age people have higher levels of MMP [7].

## 2. Reactive Oxygen Species and Photoaging

**Figure 2:**
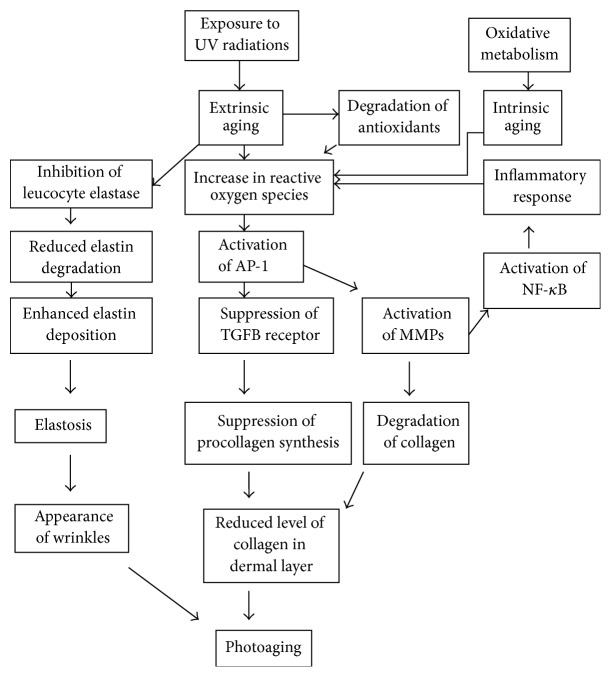
Mechanism of aging.

**Figure 3:**
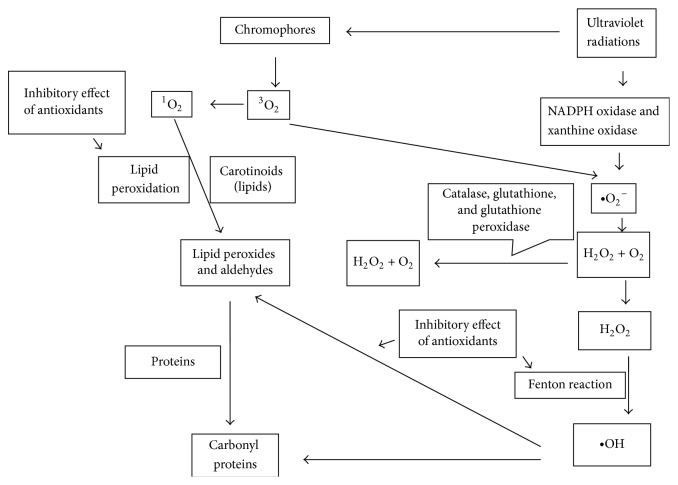
Production of ROS and its role in the initiation of oxidative chain reactions and target sites for antioxidant action.

The exposure to UVR is the main cause of oxidative stress in the skin and thus is an important risk factor for development of skin problems, for example, wrinkle formation, lesions, and cancer. On exposure to sunlight, skin molecules absorb UVR resulting in the generation of reactive oxygen species (ROS). There are two types of ROS: type 1 consists of a single, excited oxygen molecule (^1^O_2_) (Figure 3), while oxygen molecules with unpaired electron constitute second type of ROS. The examples of second type are presented in Table 1 that also describes the enzymes which are involved in the generation of these ROS [16]. Reactive oxygen entities exert a damaging effect on cellular fractions including cell walls, lipid membranes, mitochondria, nucleus, and DNA producing “oxidative stress,” that is, a difference between ROS and antioxidants, ROS being in excess leading to tissue injury and development of disease including aging, cancer, ischemia, liver injury, arthritis, and Parkinson's syndrome (Figure 2).

## 3. Benefits and Types of Antioxidants

The oxidative stress-mediated development of diseases is manageable by prolonged usage of the safe antioxidants [17]. The literature study reveals that numerous compounds have been investigated with the intention of exploring evidence against ROS-induced damage and noted their antiaging effect on skin. These compounds are efficient for overcoming sunlight-induced skin problems and making it fresh, healthy, and young through collagen synthesis [18]. Generally, the antioxidants behave as antiaging compounds in action because they are capable of scavenging ROS leaving healthy effect on skin. Since living systems have capability to maintain homeostasis of ROS in cell, the human skin is protected from UVR through complex antioxidant defense system comprising of two types of antioxidants, that is, endogenous and exogenous (consumed) antioxidants. The former category constitutes a network of protective antioxidants in skin; it includes melanin and some enzymes. Manganese-superoxide dismutase is a mitochondrial enzyme that destroys the superoxide ions produced by respiratory chain activity [19]. In general, expression of antioxidant enzymes is found very high in the epidermal layer compared to that of stratum corneum and dermis. If there is imbalance between oxidants and endogenous antioxidants, exogenous antioxidants are helpful to restore the balance. The exogenous antioxidants comprise of compounds that cannot be synthesized by human body. Vitamins, ascorbate, carotenoids, and polyphenols constitute latter type of antioxidants which are also involved in the maintenance of oxidative homeostasis [20]. The endogenous antioxidants in dermal and epidermal layers of skin exposed to sunlight are depleted under the effect of elevated levels of UVR-generated ROS. Such depletion results in the diminished activity of these antioxidants leading to skin damage [21]. With age, endogenous antioxidants are steadily consumed increasing the risk of oxidative stress; then the use of exogenous antioxidants as prevention strategy is essential. It is evident from the above discussion that skin cells are damaged by oxidative stress which might be decreased by action of the antioxidants.

## 4. Exogenous Antioxidants

The exogenous antioxidants include synthetic and natural compounds. The synthetic exogenous antioxidants include monoethanolamine, diethanolamine, sodium laureth sulfate, and triethanolamine, but these compounds have undesired effects including allergic and irritant contact dermatitis and contact dermatitis [19]. On the other hand, natural exogenous antioxidants are nontoxic in nature and produce no unwanted effect on skin.

## 5. Phytoantioxidants

**Figure 4:**
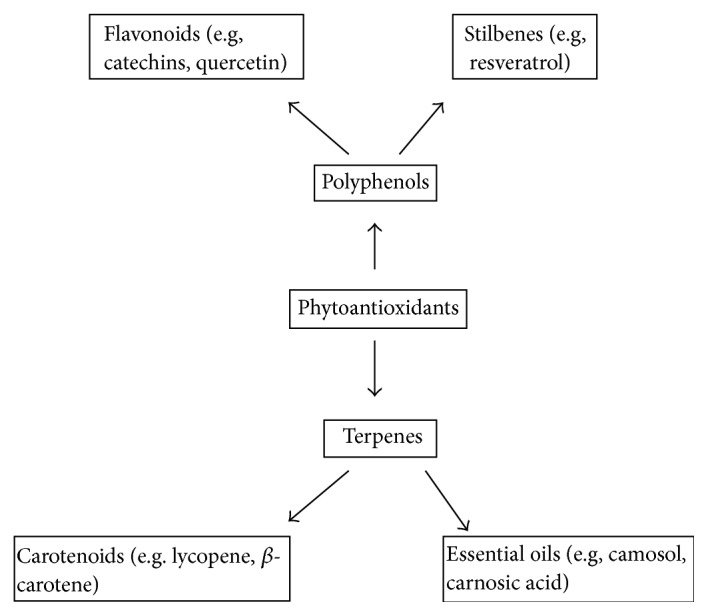
Classification of phytoantioxidants [15].

The phyto-kingdom includes vegetables, fruits, whole grains, and beverages, for example, tea, chocolate, and wine. These products are rich in natural antioxidants. An important class of natural exogenous antioxidants is phytoantioxidants, that is, antioxidants found in plants [21]. Phytoantioxidants include terpenes or polyphenols (Figure 4). After synthesis in plants, these compounds are found to have important role in the metabolism and defense system of plants. Terpenes are known to have potential for managing the oxidative stress through their free radical scavenging potential. Moreover, polyphenols occur in all parts (roots to leaves) of the plants and protect them from environmental stress through their free radical scavenging property. There are various types (>8000 phenolic structures) of polyphenols on the basis of molecular weight and polarity [64]. The structural formula of polyphenols contains phenol group(s), that is, benzene ring possessing hydroxyl group. The antioxidant activity of various polyphenols depends on number and position of phenol groups [65].

## 6. Stratum Corneum as Target Site for Antioxidants

**Figure 5:**
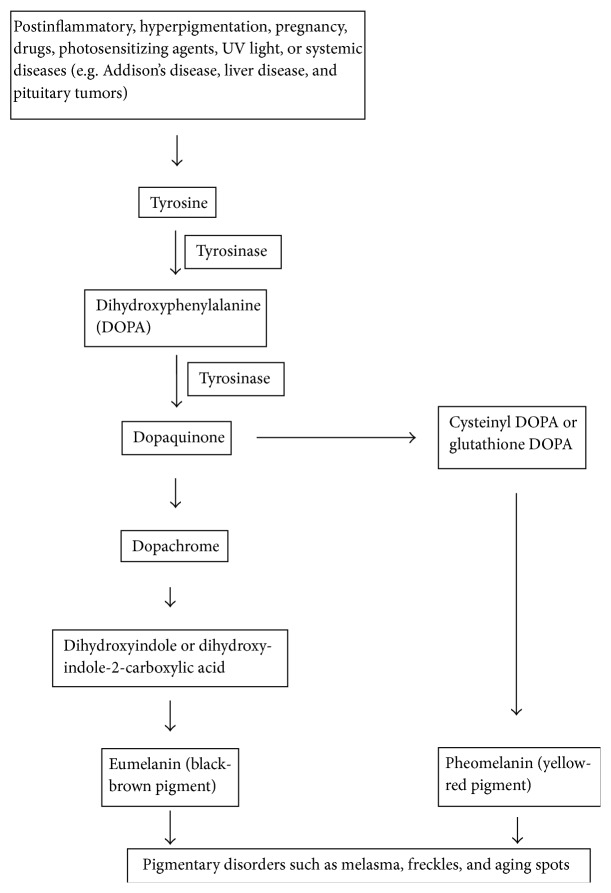
Melanin synthesis involving tyrosinase and a series of oxidative reactions.

The normal human skin maintains homeostasis of water and other materials in body, principally due to the presence of stratum corneum [66]. The stratum corneum, a water barrier in function, consists primarily of lipids, that is, ceramides, cholesterol, free fatty acids, triglycerides, stearyl esters, and cholesterol sulfate. The cholesterol sulfate is responsible for intercellular adhesion, and its high concentration is known to inhibit desquamation. The synthesis of these lipids is affected by many factors, mainly related to enzymes, fatty acids, environment, cosmetics, and water contents. Other important constituents of stratum corneum are proteins (e.g., involucrin and loricrin), enzymes, and water (approximately 30%) [67]. Depending upon natural moisturizing factor of skin cells, some fraction of this water is tightly held in stratum corneum and is responsible for skin elasticity. The disturbance in level or nature of any of lipids, proteins, enzymes, and water might lead to skin problems including wrinkled and dry skin. Dry skin might be due to excessive transepithelial water loss that could be retained for maintaining the proper skin hydration by using skin moisturizer. It might exercise softening effect on skin. The skin moisturizer, however, should be inert, nonirritant, stable, and sterile [68]. On the other hand, skin wrinkles might be due to distorted elastic fibers, diminished collagen contents, and uneven types I and III collagen. There is decrease in type IV collagen protein at the wrinkle's base; it could be due to activation of MMPs, the collagen-degrading enzymes. Alternatively, the activation of MMPs may lead to upregulation of collagenase, gelatinase, and stromelysin [69]. Thus, the skin wrinkles could be treated by using topical formulations loaded with the bioactive compounds having potential of inhibiting MMPs, thus increasing the collagen level. Moreover, skin colour depends on the kind and allocation of melanin in the skin, in addition to number and amount of melanocytes [67, 70].  Figure 5 shows the melanin synthesis involving tyrosinase and a series of oxidative reactions that could be inhibited by usage of skin-whitening agents. Thus, the stratum corneum is a primary target site for topical phytoantioxidants for skin protection against UVR-mediated oxidative stress [71]. The phytoantioxidants might have capability of stimulating the regeneration of stratum corneum to protect itself and the underlying epidermis and dermis from the injurious effects of UVR and promote growth of the skin [72, 73].

## 7. Skin Care Products

The pharmaceutical formulations used for skin care, termed as the cosmetics, could be herbal in nature. The herbal cosmetics might contain the isolated bioactive compounds or the crude phytoextracts [74]. Currently, there are extensive research activities in progress involving development and characterization of extract loaded formulations to concurrently achieve various goals such as anti-inflammatory and antiaging effect [75]. There are three types of bioactive compounds present in various phytoextracts; the compounds include polyphenols, flavonoids, and carotenoids. These compounds exert both the antioxidant and the UV protection effect [76].

## 8. Pharmaceutical Creams

Skin care products could be solid, semisolid, or liquid. The semisolid formulations include creams, ointments, and pastes. Cream is an emulsion of oil and water, prepared for skin applications [77]. Emulsions represent a class of disperse systems which comprise of two insoluble, thermodynamically stable phases, that is, continuous and dispersed phase [78]. The emulsion is water-in-oil if the dispersed phase is oil and vice versa. This type of emulsion is termed as simple emulsion. If simple emulsion is further dispersed in the dispersed phase medium, such type of system is termed as the multiple emulsion. Based on globule size of dispersed phase, emulsions can be grouped into various classes including macro- and microemulsion [79]. Emulsions constitute an exclusive class of cosmetics that produce a pleasant feeling to skin on application, acceptable for long-term use, improved spreadability of the ingredients, and remain stable during long storage period [80]. Owing to these characteristics, emulsions are extensively used as a vehicle in drug delivery, particularly across the skin. In particular, for dry skin, water-in-oil (W/O) emulsions are more broadly used for the treatment of dermatological concerns [81]. The addition of antioxidants as active ingredients endows these emulsions with features of cosmetics. For improved cosmetic features, the botanical extracts can be added to the cosmetic creams since the extracts comprise of a number of antioxidants that might produce synergistic effect [82].

## 9. Preparation and Characterization of Phytoextract Loaded Creams

The creams are topically used to protect and treat the skin problems including hyperdepigmentation and wrinkles. Beside these advantages, the creams may produce skin problems such as infection, photosensitivity, erythema, contact dermatitis, cancer, and/or change in skin colour. During the development of such antiaging creams, the researchers should be more focused on the elucidated sources, structures, and interactive modes of the composite active constituents with the skin to achieve maximum formulation efficacy and skin safety [83].

The preparation of herbal creams may involve the modified methodology using isolated phytochemicals or the extracts along with appropriate composition of the mandatory constituents essentially employed for creams with desirable features [84]. Due to technical sophistication of cream development, it is accentuated that the phytochemicals maintain their bioactivity during extreme processing. To ensure effective and stable formulations, exhaustive analytical strategies are adopted. Various physicochemical characterization parameters include stability, pH, and viscosity testing [85].  Table 3 shows various types of equipment used for* in vivo* characterization of botanical creams.

## 10. Phytoextract Loaded Creams

Due to the presence of numerous bioactive ingredients in phytoextracts, extract loaded creams are considered more efficacious with lesser side effects against aging in comparison to creams loaded with specific individual antioxidant. Owing to tremendous antioxidant potential, phytoextracts are extensively used in numerous cream formulations. Up to now,* Acacia nilotica, Benincasa hispida, Calendula officinalis, Camellia sinensis, Nelumbo nucifera, Capparis decidua, Castanea sativa, Coffea arabica, Crocus sativus, Emblica officinalis *Gaertn,* Foeniculum vulgare, Hippophae rhamnoides, Lithospermum erythrorhizon, Malus domestica, Matricaria chamomilla* L.,* Moringa oleifera, Morus alba, Ocimum basilicum, Oryza sativa, Polygonum minus, Punica granatum, Silybum marianum, Tagetes erecta *Linn.,* Terminalia chebula, Trigonella foenum-graecum*, and* Vitis vinifera *have successfully been used in developing the stable cream formulations with excellent antioxidant effect, possibly due to presence of multiple antioxidant phytochemicals. In this review article, the documented phytoextract loaded creams with their characterization on human skin have been discussed with special emphasis on their bioactive constituents (Table 2).

### 10.1. *Acacia nilotica*

*Acacia nilotica* (Mimosaceae) contains tannins, gallic acid, phlobatannin, pyrocatechol, (+)-catechin, protocatechuic acid, (−)-epigallocatechin-7-gallate, and (−)-epigallocatechin-5, 7-digallate. All parts of this plant, from root to flowers, possess various medicinal activities such as antidiabetic, antiasthmatic, and anticancer [86]. Ali et al. prepared water-in-oil cream of bark extract of* Acacia nilotica* and applied it to photoaged skin [27]. They found improved mechanical features of skin, that is, reduced levels of roughness, scaliness, smoothness, and wrinkles of photoaged skin. The investigators attributed this antiaging activity to the phenolic compounds present in extract; the phenolics have capability to quench ROS.

### 10.2. *Benincasa hispida*

The major constituents of* Benincasa hispida* (Cucurbitaceae) are triterpenoids, flavonoids, glycosides, saccharides, carotenes, vitamins, *β*-sitosterin, and uronic acid [87]. The fruit of this plant is effective for different diseases including cardiac disease, diabetes, inflammation, and cancer [88]. The water-in-oil cream of fruit extract of* Benincasa hispida* showed antioxidant activity revealing its potential to retard symptoms of aging [28].

### 10.3. *Calendula officinalis*

*Calendula officinalis* belongs to family Compositae. This plant is rich in active compounds including terpenoids, carotenoids, flavonoids, and volatile oils [89]. The water-in-oil cream of flower extract of* Calendula officinalis* exhibited aptitude of stimulating skin tightness and improved skin elasticity leading to delayed aging process. Moreover, this preparation enhances the skin hydration level, as evident from reduced TEWL values, which is crucial for normal cutaneous metabolism; it prevents early aging [29]. In addition, the reduction in skin melanin contents and the decrease in skin sebum level were also observed after application of this formulation [30].

### 10.4. *Camellia sinensis* (Green Tea) and* Nelumbo nucifera* (Lotus)

Potent antioxidants have been isolated from both green tea and lotus. Green tea is rich in a polyphenol, catechins, especially EGCG which is a strong antioxidant [90]. The water-in-oil cream loaded with ethanolic leaf extract of* Camellia sinensis* significantly reduced the sebum production as compared to base formulation (formulation without extract) [31]. In another study, Mahmood et al. reported reduced TEWL values and thus improved skin hydration level using this formulation [32]. Furthermore, the W/O/W nano-multiple emulsion containing alcoholic extracts of* Camellia sinensis* (Theaceae) leaves and* Nelumbo nucifera* (Nelumbonaceae) plant, alone or in combination, was formulated and tested on human skin. Both extracts were found to have diminished sebum secretions for mono (green tea or lotus) and combined treatments (green tea plus lotus) after applying same formulations to volunteers' skin. Green tea plus lotus together produced statistically better antisebum effect [33]. It indicates that active ingredients in lotus add a synergistic effect to the activity of green tea that can be attributed to 5*α*-reductase inhibition activity of both extracts [91]. Zinc compounds, polyphenolics, flavonoids, tannic acid, and linoleic acid are excessively found in lotus [92]. Linoleic acid, a polyunsaturated fatty acid, and EGCG have an advantage of inhibiting sebum production due to their 5*α*-reductase inhibition activity [93]. In another study, the researchers noted the significant effectiveness of nano-multiple emulsion against skin wrinkles, roughness, and scaliness leading to skin revitalization effect. Green tea and lotus combined in multiple emulsions exerted a superior synergistic antiaging effect [34]. In addition, Mahmood and Akhtar also reported that this formulation had an advantage of reducing melanin and improving hydration contents of skin without causing erythema [35].

### 10.5. *Capparis decidua*

*Capparis decidua *belongs to family Capparidaceae [94]. This plant is rich in active compounds including isothiocyanate glucoside, glucocapparin, stachydrine, n-triacontane, *β*-carotene, and *β*-sitosterol [95]. The water-in-oil emulsion cream of plant extract of* Capparis decidua* exhibited reduction in skin sebum level after application on skin [36].

### 10.6. *Castanea sativa*

*Castanea sativa* (Fagaceae) contains catechin, myricetin 3-O-glucoside, quercetin 3-O-rutinoside, quercetin 3-O-glucoside, kaempferol 3-O-rutinoside, and kaempferol 3-O-glucoside. The surfactant-free cream of ethanolic extract of* Castanea sativa* leaves has been found to produce moisturizing effect on human skin by controlling transepithelial water loss [37].

### 10.7. *Coffea arabica*

*Coffea arabica* belongs to family Rubiaceae. This coffee plant is rich in active compounds including chlorogenic acid, condensed proanthocyanidins, quinic acid, and ferulic acid [96]. All of these polyphenolic compounds possess antioxidant property [97]. McDaniel prepared cream of berry extract of* Coffea arabica* and found reduced levels of MMP-1 and IL-1b [38]. Moreover, upregulated gene expression for four collagen structural proteins and downregulated gene expression for three MMPs were also observed concluding reparative effects of* Coffea arabica* extract upon photoaged skin.

### 10.8. *Crocus sativus*

Water-in-oil emulsion based cream loaded with ethanolic extract of* Crocus sativus* flowers has been found to produce moisturizing effect on human skin by controlling transepithelial water loss [39].* Crocus sativus* (Iridaceae) contains zeaxanthin, lycopene, carotenes, crocetin, and picrocrocin.

### 10.9. *Emblica officinalis* Gaertn

Some potent antioxidants including gallotannins, for example, emblicanin A, emblicanin B, punigluconin, and pedunculagin, are present in* Emblica officinalis *Gaertn (Euphorbiaceae), generally known as Amla [98]. This plant exists in China, India, and Indonesia. All parts of this plant, from root to fruit, are medicinally effective in different health problems; for example, diarrhea and jaundice are effectively treated by using its fruit [99]. The hydroalcoholic fruit extract of* Emblica officinalis* has been formulated as water-in-oil cream that is found effective for reducing transepidermal water loss, as observed by using Tewameter [40]. Since transepidermal water loss plays crucial role in aging process this formulation can be employed as an antiaging product. This property of cream could be due to presence of strong antioxidants in* Emblica officinalis.*

### 10.10. *Foeniculum vulgare*

On application of water-in-oil emulsion based cream loaded with ethanolic extract of* Foeniculum vulgare* (Apiaceae) seeds for eight months, reduced transepithelial water loss was observed which resulted in improved moisture contents on human skin [41]. Moreover, there was an improvement in skin mechanical properties, that is, reduced levels of roughness, scaliness, smoothness, and wrinkles of photoaged skin. In another study, Rasul et al. applied same formulation on hyperpigmented human skin and observed the decreased melanin level, sebum production, and erythema of the treated skin [42]. The decrease in skin melanin level leads to skin-whitening effect. Beside flavonoids, they attributed this effect to linoleic acid present in the used extract. This unsaturated fatty acid, a main constituent of biological cell membranes, has an advantage of accelerating process of tyrosinase degradation resulting in reduced melanin synthesis due to low tyrosinase levels [100]. Furthermore, this cream may be useful for skin acne due to its diminishing effect on skin sebum level. Oleic acid, linolenic acid, and linoleic acid, present in* Foeniculum vulgare* extract, could be responsible for this effect [101]. These unsaturated fatty acids have inhibitory effect on sebum production owing to selective inhibition of 5-reductase which is involved in production of sebum [102]. Lastly, there was decrease in erythema on treated skin; this effect elaborates the anti-inflammatory action of this cream.

### 10.11. *Hippophae rhamnoides*

By using Cutometer, Khan et al. reported the improvement in facial skin mechanical parameters, for example, skin elasticity, indicating antiaging effect after using water-in-oil based hydroalcoholic cream loaded with fruit extract of* Hippophae rhamnoides* [43]. Another study reported the antisebum secretion effect of same formulation [103]. Moreover, the extracts of* Hippophae rhamnoides* and* Cassia fistula* were also found effective in the reduction of skin sebum content (antiacne effects) in human with grade I and grade II acne vulgaris [104]. Khan et al. reported that this formulation improves barrier function of human skin as tested by Tewameter and Corneometer [105]. The possible antiaging effect was pointed out as a feature of antioxidants such as carotene, particularly *β*-carotene, vitamin C, and vitamin E present in extract. Vitamin C occurs in a concentration of 28–2500 mg/100 g of* Hippophae rhamnoides* extract [106] and plays a role in the stimulation of dermal fibroblasts to synthesize collagen which is responsible for holding water contents in skin [107]. The* Hippophae rhamnoides* extract affects skin mechanical properties through increased expression of cell surface integrins that promote collagen contraction. Moreover, Khan et al. reported reduced TEWL and thus increased skin hydration level for same formulation [108]. Furthermore, same researchers also described reduction in skin melanin level and erythema by using this cream [109].

### 10.12. *Lithospermum erythrorhizon*

Oil-in-water emulsion based cream loaded with ethanolic extract of* Lithospermum erythrorhizon* root has been found to produce moisturizing effect on human skin by controlling transepithelial water loss.* Lithospermum erythrorhizon* (Boraginaceae) contains shikonin, acetylshikonin, deoxyshikonin, b-acetoxyisovalerylshikonin, isobutylshikonin, b,b-dimethyl acrylshikonin, 2-methyl-n-butyrylshikonin, and isovalerylshikonin [44].

### 10.13. *Malus domestica*

The reduced transepithelial water loss has been observed leading to improved moisture contents in human skin after application of water-in-oil emulsion based cream loaded with hydroalcoholic extract of* Malus domestica* (Rosaceae) seeds for eight months [45]. Moreover, there was an improvement in skin mechanical properties, that is, reduced levels of roughness, scaliness, smoothness, and wrinkles of the photoaged skin. In another study, Khan et al. applied same formulation on the hyperpigmented human skin and observed decreased melanin level, sebum production, and erythema of the treated skin. These antiaging effects on skin could be attributed to flavonoid, quercetin, and hesperetin, present in* Malus domestica* extract [46].

### 10.14. *Matricaria chamomilla* L

After skin treatment with water-in-oil emulsion based cream loaded with hydroalcoholic extract of* Matricaria chamomilla* plant for eight weeks, reduced transepithelial water loss was observed. Since an increase in transepithelial water loss shows disruption of the stratum corneum and loss of intercellular lipids, accordingly this formulation possibly repairs the stratum corneum and improves the moisture contents in human skin [47]. Moreover, there was an improvement in skin mechanical properties, that is, diminished levels of roughness, scaliness, smoothness, and wrinkles of photoaged skin [110, 111]. The investigators attributed this antiaging activity to the phenolic compounds present in extract.* Matricaria chamomilla* belongs to Asteraceae family and contains some bioactive ingredients including terpenes, polysaccharides, and flavonoids such as *α*-bisabolol and apigenin.

### 10.15. *Moringa oleifera*

*Moringa oleifera* (Moringaceae) contains carotene, vitamin C, vitamin B, vitamin A, carotenoids, myricetin, quercetin, kaempferol, gallic acid, syringic acid, and rutin. All parts of this plant, from root to leaves, possess various medicinal activities such as antibacterial, anticancer, and antioxidant [112, 113]. Ali et al. prepared water-in-oil cream of hydroalcoholic extract of* Moringa oleifera* leaves and applied it to photoaged skin [48, 49]. The investigators found reduced undesirable skin sebum contents of skin and diminished skin transepidermal water loss leading to increased skin hydration, particularly for dry skin using Sebumeter and Corneometer, respectively. In addition, same formulation was also found effective against skin wrinkles, roughness, and scaliness leading to skin revitalization effect [50]. They tagged the antiaging characteristic of* Moringa oleifera* to the phenolic compounds present in extract, since the phenolics have capability to scavenge ROS.

### 10.16. *Morus alba*

*Morus alba* belongs to family Moraceae. This plant is rich in active compounds including anthocyanins, gallic acid, flavonoids and tannins, citric acid, vitamin C, and palmitic acid [114, 115]. Akhtar et al. prepared oil-in-water cream of hydroalcoholic extract of* Morus alba* fruit followed by application to the photoaged skin of the human volunteers [53]. After 8 weeks, the studied skin areas were tested using Mexameter and Corneometer. The results indicated the reduction in melanin contents of skin, without producing erythema, attributing this activity to the presence of anthocyanin and flavonoids. These phenolics have tyrosinase inhibition activity, one of the modes of antiaging activity [51, 52, 116]. In another study, Akhtar et al. reported decrease in erythema and melanin contents in the treated skin using same formulation [52].

### 10.17. *Ocimum basilicum*

Rasul and Akhtar reported the improvement in facial skin mechanical (viscoelasticity) and biochemical parameters (superoxide dismutase, catalase, total protein, and ascorbic acid level) when skin was treated with water-in-oil emulsion based cream loaded with ethanolic extract of* Ocimum basilicum *(Lamiaceae) seeds [53]. Moreover, the reduction in malondialdehyde level was also noted. The possible antiaging effect was pointed out as a feature of antioxidants such as quercetin, isoquercetin, kaempferol, caffeic acid, rosmarinic acid, rutin, catechin, ferulic acid, rutinoside, and apigenin present in extract [117].

### 10.18. *Oryza sativa*

Some strong antioxidants including ferulic acid, gamma-oryzanol, and phytic acid are present in* Oryza sativa* (Poaceae). The grains extract of* Oryza sativa* was loaded to niosomes followed by the preparation of water-in-oil cream using these niosomes. This cream was found effective for reducing transepidermal water loss, as observed by using Corneometer and Evaporimeter. Since transepidermal water loss plays crucial role in aging process by enhancing skin hydration, this formulation can be employed as an antiaging product. This feature of cream could be due to strong antioxidants present in* Oryza sativa* extract [54].

### 10.19. *Polygonum minus*

Haris et al. reported the improvement in facial skin elasticity as well as reduced wrinkles after treating with water-in-oil emulsion based cream loaded with aqueous extract of* Polygonum minus* (Polygonaceae) seeds [55]. The possible antiaging effect could be due to the antioxidants such as caffeic acid and quercetin [118].

### 10.20. *Punica granatum*

Kaur and Saraf reported the improvement in facial skin mechanical (viscoelasticity) and biochemical parameters (catalase and ascorbic acid concentration) [56]. Moreover, the reduction in malondialdehyde level was also noted. These results indicate the antiaging effect of nanotransfersomes loaded cream. The investigators used nanotransfersomes loaded with ethanolic extract of* Punica granatum* (Punicaceae) seeds to prepare a novel cream beside development of conventional cream. The antiaging effect of various formulations was in this decreasing order: nanotransfersomal cream > conventional cream > blank nanotransfersomal cream > base cream. The possible antiaging effect was pointed out as a feature of antioxidants such as anthocyanins, ellagic acid, and hydrolysable tannins present in extract.

### 10.21. *Silybum marianum*

On application of water-in-oil emulsion based cream loaded with ethanolic extract of* Silybum marianum* (Asteraceae) seeds for eight months, reduced transepithelial water loss was observed which resulted in improved moisture contents in human skin [57]. Moreover, there was an improvement in skin mechanical properties, that is, reduced levels of roughness, scaliness, smoothness, and wrinkles of photoaged skin. In another study, Rasul et al. applied same formulation on hyperpigmented human skin and observed the decreased melanin level, sebum production, and erythema of the treated skin [58].

### 10.22. *Tagetes erecta* Linn

*Tagetes erecta *Linn. (Asteraceae) contains quercetagetin, syringic acid, lutin, quercetin, and gallates. This plant possesses different medicinal activities including antiaging, anticancer, and anti-inflammatory [119–121]. Leelapornpisid et al. prepared ethyl acetate extract of* Tagetes erecta* flowers and loaded it to nanostructured lipid carriers [59]. These carriers were then formulated as cream and applied to photoaged skin. Using Visiometer, the researchers observed reduction in skin wrinkles of photoaged skin without producing skin irritation after eight weeks of cream usage. The investigators attributed this antiaging activity to the antioxidants present in extract.

### 10.23. *Terminalia chebula*

After skin treatment with water-in-oil emulsion based cream loaded with hydroalcoholic extract of* Terminalia chebula* plant for eight weeks, reduced transepithelial water loss was observed. Moreover, there was decrease in skin melanin contents also [60]. These features could be due to the phenolic compounds present in extract.* Terminalia chebula* belongs to Combretaceae family and contains some bioactive ingredients including gallic acid, ellagic acid, tannic acid, ethyl gallate, chebulic acid, chebulagic acid, corilagin, and ascorbic acid [122].

### 10.24. *Trigonella foenum-graecum*

*Trigonella foenum-graecum* belongs to family Fabaceae. This medicinal plant is rich in active compounds including polyphenols, galactomannans and flavonoid, protodioscin, trigoneoside, diosgenin, and yamogenin. Galactomannan has an advantage of improving skin hydration [123]. Waqas et al. and Akhtar et al. prepared water-in-oil cream of methanol extract of* Trigonella foenum-graecum* seeds [61, 62]. After applying this cream to human skin for predetermined time, the former authors observed an improvement in facial skin mechanical parameters without producing erythema, while the latter authors reported reduction in skin melanin contents and maintenance of skin hydration.

### 10.25. *Vitis vinifera*

Various bioactive compounds such as sarmentine are present in* Vitis vinifera* (Vitaceae). The hydroalcoholic shoot extract of* Vitis vinifera *was formulated as water-in-oil cream that was found effective for improving clinical signs of photoaged skin. This property of cream could be due to the presence of strong antioxidants in* Vitis vinifera* extract [63].

## 11. Conclusion

Due to constant exposure of human skin to the UV radiations present in sunlight, several pathobiological alterations in cells occur. The photoprotection is the main approach for managing the photoaging, but cosmeceuticals could also be used as an alternative therapy. The selection of therapeutic approaches depends on the nature of these injurious molecular changes. Large number of botanical extract loaded creams have been prepared and assessed (Table 1) for their antiaging potential. The observed antiaging effects of cream formulations could be an outcome of a coordinating action of multiple constituents. Of numerous botanicals, the phenolic acids and flavonoids appear effective against UV radiation-induced damage; however, the evidence-based studies for their antiaging effects are still needed. Since environment affects the skin-cream interaction, the cautious assessment of their clinical efficacy should be conducted in future.

## Figures and Tables

**Table 1:** Enzymes involved in the generation of ROS with an unpaired electron.

Number	Name	Symbol	Enzymes
1	Superoxide anion radicals	^•^O_2_^−^	NADPH oxidase and xanthine oxidase [22–24]
2	Hydroxyl radicals	^•^OH	Superoxide dismutase (SOD) [25]
3	Nitric oxide radicals	NO^•^	Nitric oxide synthase (NOS) [26]
4	Lipid peroxyl radicals	LOO^•^	Superoxide dismutase [25]

**Table 2:** Phytoextracts loaded to creams with antioxidant features studied in human.

Botanical name	Family	Part used	Nature of extracts	Antioxidants^†^	Nature of cream	Oil phase	Emulsifier	References
*Acacia* *nilotica*	Mimosaceae	Bark	Ethanol	Phlobatannin, pyrocatechol, (+)-catechin, protocatechuic acid, (−)-epigallocatechin-7-gallate, and (−)-epigallocatechin-5, 7-digallate	Simple W/O cream^††^	Paraffin oil	ABIL EM 90	[27]

*Benincasa hispida*	Cucurbitaceae	Fruit	Petroleum ether	Caffeic acid	Simple W/O cream^††^	Cetyl alcohol	Polysorbate	[28]

*Calendula* *officinalis*	Compositae	Flowers	Ethanol	Isorhamnetin, quercetin, myricetin, and kaempferol	Simple W/O cream^††^	Paraffin oil	ABIL EM 90	[29, 30]

*Camellia sinensis*	Theaceae	Leaves	Ethanol	Epigallocatechin gallate	Simple W/O cream^††^	Paraffin oil	ABIL EM 90	[31, 32]

*Camellia sinensis *(green tea) and* Nelumbo nucifera *(lotus)	*Camellia sinensis* (Theaceae): *Nelumbo nucifera *(Nelumbonaceae)	Leaves of *Camellia sinensis*: whole plant material of *Nelumbo nucifera*	Ethanol for *Camellia sinensis*; methanol for *Nelumbo nucifera*	Epigallocatechin gallate in green tea; hyperin, isoquercetin, and astragalin in lotus	W/O/W nano-multiple-emulsions	Paraffin oil	ABIL EM 90, polyoxyethylene (20) cetyl ether, Cetomacrogol 1000	[33–35]

*Capparis decidua*	Capparidaceae	Full plant	Methanol	Isoginkgetin and ginkgetin	Simple W/O cream^††^	Paraffin oil	ABIL EM 90	[36]

*Castanea sativa*	Fagaceae	Leaves	Ethanol	Catechin and myricetin derivatives	Simple W/O cream^††^		Surfactant-free formulation	[37]

*Coffea* *arabica*	Rubiaceae	Berry	Ethanol	Chlorogenic acid, condensed proanthocyanidins, quinic acid, and ferulic acid	Simple cream^††^	Information not available	Information not available	[38]

*Crocus sativus*	Iridaceae	Flowers	Ethanol	Zeaxanthin, lycopene, carotenes, crocetin, and picrocrocin	Simple W/O cream^††^	Paraffin oil	ABIL EM 90	[39]

*Emblica officinalis *Gaertn	Euphorbiaceae	Fruit	Hydroalcoholic	Emblicanin A, emblicanin B, punigluconin, and pedunculagin	Simple W/O cream^††^	Paraffin oil	ABIL EM 90	[40]

*Foeniculum vulgare*	Apiaceae	Seeds	Ethanol	Gallic acid, caffeic acid, ellagic acid, quercetin, and kaempferol	Simple W/O cream^††^	Paraffin oil	ABIL EM 90	[41, 42]

*Hippophae* *rhamnoides*	Elaeagnaceae	Fruit	Hydroalcoholic	Isorhamnetin, quercetin, myricetin, and kaempferol	Simple W/O cream^††^	Paraffin oil	ABIL EM 90	[43]

*Lithospermum erythrorhizon*	Boraginaceae	Root	Ethanol	Naphthoquinone (shikonin, acetylshikonin, deoxyshikonin, b-acetoxyisovalerylshikonin, isobutylshikonin, b,b-dimethyl acrylshikonin, 2-methyl-n-butyrylshikonin, and isovalerylshikonin)	Simple O/W cream^††^	Cyclomethicone, caprylic/capric triglyceride, phytosphingosine (0.005%), and cholesterol	Sodium lauroyl lactylate	[44]

*Malus domestica*	Rosaceae	Fruit	Methanol : formic acid : double distilled water (70 : 2 : 28)	Hesperetin	Simple W/O cream^††^	Paraffin oil	ABIL EM 90	[45, 46]

*Matricaria chamomilla *L.	Asteraceae	While plant	Hydroalcoholic	*α*-Bisabolol and apigenin	Simple W/O cream^††^	Cetyl alcohol	Sodium lauroyl lactylate	[47]

*Moringa oleifera*	Moringaceae	Leaves	Hydroalcoholic	Epigallocatechin gallate, myricetin, quercetin, rutin, morin, taxifolin, chrysin, baicalein, fisetin, biochanin A, genistein, kaempferol, emodin anthraquinone, caffeic acid phenethyl ester, and octyl and dodecyl gallates	Simple O/W cream^††^	Paraffin oil	ABIL EM 90	[48–50]

*Morus alba*	Moraceae	Fruit	Hydroalcoholic	Rutin, quercetin, isoquercitrin, and quercetin	Simple O/W cream^††^	Paraffin oil	ABIL EM 90	[51, 52]

*Ocimum basilicum*	Lamiaceae	Seeds	Ethanol	Quercetin, isoquercetin, kaempferol, caffeic acid, rosmarinic acid, rutin, catechin, ferulic acid, rutinoside, and apigenin	Simple W/O cream^††^	Paraffin oil	ABIL EM 90	[53]

*Oryza sativa*	Poaceae	Grains	Ethanol	Gallic acid, pyrogallol, apigenin, and rutin	Niosomes loaded cream	Information not available	Information not available	[54]

*Polygonum minus*	Polygonaceae	Leaves	Aqueous	Caffeic acid and quercetin	Simple W/O cream^††^	Isoparaffin	Laureth-7	[55]

*Punica granatum*	Punicaceae	Seeds	Ethanol	Ellagic acid	Nanotransfersomes loaded cream	Cetyl alcohol	Span 60 and tween 80	[56]

*Silybum marianum*	Asteraceae	Seeds	Ethanol	Silymarin (silybin, silydianin, and silychristin)	W/O emulsion cream	Paraffin oil	ABIL EM 90	[57, 58]

*Tagetes erecta* Linn.	Asteraceae	Flowers	Ethyl acetate	Lutein	Nanostructured lipid carrier loaded cream	Glyceryl monostearate, stearic acid, octyldodecanol, and mineral oil	Tween, span, or triethanolamine stearate	[59]

*Terminalia chebula*	Combretaceae	Seeds	Methanol	Gallic acid, ellagic acid, tannic acid, ethyl gallate, chebulic acid, chebulagic acid, corilagin, and ascorbic acid	Simple W/O cream^††^	Paraffin oil	ABIL EM 90	[60]

*Trigonella foenum-graecum*	Fabaceae	Seeds	Methanol	Kaempferol derivatives such as 3-O- -D-glucosyl(1 2) - -D-galactoside	Simple W/O cream^††^	Paraffin oil	ABIL EM 90	[61, 62]

*Vitis vinifera*	Vitaceae	Shoot	Ethanol	Resveratrol, delphinidin, peonidin, petunidin, malvidin, and (+)-catechin	Simple W/O cream^††^	Cetyl alcohol	Span 60	[63]

^†^Including but not limited to.

^††^Simple cream is the cream that is not loaded with some novel carrier system such as nanotransfersomes.

**Table 3:** Equipment used for *in vivo* characterization of botanical creams.

Number	Equipment	Purpose of use
1	Mexameter	Erythema analysis
2	Tewameter	Transepidermal water loss (TEWL) evaluation
3	Corneometer	Detection of skin hydration
4	Evaporimeter	Barrier function test
5	Sebumeter	Assessment of skin surface sebum/lipid contents
6	Visiometer	Wrinkle test
7	Cutometer	Measurement of skin mechanical properties/elasticity
8	Chromameter	Skin colour test
